# Supplementary material for: Contraceptive use among reproductive-age females with disabilities in central Sidama National Regional State, Ethiopia: a multilevel analysis
Source: PeerJ. 2023 May 12;11:e15354. doi: 10.7717/peerj.15354 (PMC10184657; doi:10.7717/peerj.15354)
Supplement: Supplemental Information 2 [file peerj-11-15354-s002.docx]

## **Participant information**

My name is Zelalem Tenaw Currently I am a PhD. Student at Hawassa University in public health and now I am researching reproductive health services (RHS) among people with disabilities (PWDs) in Dale and Wonsho districts, and Yirgalem city administration, Sidama National Regional State.

**Objective:** To provide new knowledge that could support the efforts to improve the RHSU of reproductive-age PWDs in SNRS, Ethiopia. You are selected randomly as a possible participant in this study.

**Potential risks:** There is no potential risk that may cause any harm to study participants.

**Benefits:** No financial benefits are related to this study. But by participating in this study, you contribute to improving reproductive health service utilization among PWDs.

**Confidentiality:** Your name will not be written in this form and will never be used in connection with any information you tell us. All information given by you will be kept strictly confidential. Your participation is voluntary and you are not obligated to answer any question which you do not wish to answer. If you feel discomfort responding to the questionnaire, please feel free to drop it. This questionnaire will take about 20 minutes.

Are you willing to participate in the study?

1- Yes 2 - No

If the answer is yes, thanks! Conduct the interview.

If the answer is no, Thanks! Don’t force or reinforce an individual to participate in the study

**Written consent form participants/family members/head of the household**

**Hawassa University**

I have read/heard and understood all about the objective and the process of the study. My participation is voluntary and not obligated to answer any question which I do not know or do not wish to answer. I also understood that all information given by me will be kept strictly confidential. Therefore I am willing to participate in this study.

Study participant sign _____________________________ date _____________

Data collector name: _________________________________________Signature _________

Date of interview ----------- Month --------------- /2014 E. C.

Supervisor’s Name ___________________________________________ Signature________

Checked date________________ /2014E.C.

Complete 1

Incomplete 2

Contact Address of the Principal investigator

Name: Zelalem Tenaw

E-Mail: [abigiatenaw@gmail.com](mailto:abigiatenaw@gmail.com)

Cell-Phone: +251-916415147

**Section I. Respondent’s socio-demographic characteristics**

This questionnaire is designed to collect information from respondents concerning reproductive health services and problems among PWDs in Sidama Regional State, Ethiopia.

Wereda: _________________ Kebele code: _________Participant code: ____________

| NO | Question | Response | | | Skip |
| --- | --- | --- | --- | --- | --- |
| 201 | Sex | 1. Male 2. Female | | |  |
| 202 | How old are you? | ____years ( in completed year) | | |  |
| 203 | What is your religion? | 1. Orthodox 2. Catholic 3. Protestant 4. Muslim 5. Other (Specify)___________ | | |  |
| 204 | What is your ethnic group | 1. Sidama 2. Amhara 3. Oromo 4. Wolayita 5. Guragie 6. Others (specify)_____________ | | |  |
| 205 | What is your marital status | 1. Never married 2. Married 3. Divorced/separated 4. Widowed | | |  |
| 206 | Residence | 1. Urban 2. Rural | | |  |
| 207 | Kebele | Mention: _____________________ | | |  |
| 208 | With whom you are living now? | 1. Husband/wife 2. Family member 3. Alone 4. Relatives 5. Friends/peers 6. Others (specify)__________________ | | |  |
| 209 | What is your educational status? | 1. Unable to read and write 2. Attended formal education | | |  |
| 210 | If attended formal education, specify the highest grade completed | ________Grade | | |  |
| 211 | What is your employment status? | 1. Employed 2. Not employed-------------------- | | | 213 |
| 212 | Types of employer | 1. Government 2. Non-governmental organizations (NGO) 3. Family members 4. Non-family member 5. Self-employed | | |  |
| 213 | Do you have an occupation? | 1. Yes 2. No ---------------------------------- | | | 215 |
| 214 | What is your occupation? | 1. Professional/technical/managerial 2. Clerical/religious 3. Sales and services 4. Skilled manual 5. Unskilled manual 6. Agriculture 7. Other (specify)______________ | | |  |
| 215 | What is your view about yourself? (Self-perception? | 1. Good self-perception 2. Bad self-perception 3. Not sure | | |  |
| 216 | Do you have community based health insurance? | 1. Yes 2. No ----------------------------------- | | | 301 |
| 217 | Mention types of community based health insurance | ______________________________ | | |  |
| **Section II: Participants and household wealth index:** Ask (and observe when possible) the following questions one at a time and write the response on the space provided or encircle the appropriate response from the options listed. | | | | |  |
| 301 | What is the main source of drinking water for members of your household? | Piped water:   - 1. Piped into dwelling   2. Piped to yard/plot   3. Public tab/standpipe   4. Borehole | | |  |
|  |  | Dug well:   1. Protected well 2. Unprotected well | | |  |
|  |  | Water from spring:   1. Protected spring 2. Unprotected spring | | |  |
|  |  | Surface water:   - - - 1. River/lake/pond/stream/dam | | |  |
| 302 | What is the main source of water used by your household for other purposes such as cooking and handwashing? | Piped water:   1. Piped into dwelling 2. Piped to yard/plot 3. Public tab/standpipe 4. Borehole | | |  |
|  |  | Dug well:   1. Protected well 2. Unprotected well | | |  |
|  |  | Water from spring:   1. Protected spring 2. Unprotected spring | | |  |
|  |  | Surface water:   1. River/lake/pond/stream/dam | | |  |
| 303 | Where is that water source located? | 1. In own dwelling  2. In own yard/plot  3. Elsewhere | | |  |
| 304 | How long does it take to go there, get water, and come back? | 1. Minutes __________ 2. Don’t know | | |  |
| 305 | In the past two weeks, was the water from this source not available for at least one full day? | 1. Yes 2. No 3. Don’t know | | |  |
| 306 | Do you do anything to the water to make it safer to drink? | 1. Yes 2. No ------------------------------------- | | | 308 |
| 307 | What do you usually do to make the water safer to  Drink?  Record all mentioned | 1. Boil 2. Add bleach/chlorine 3. Strain through a cloth 4. Use water filter (Sand/composite/etc.) 5. Solar disinfection 6. Let it stand and settle | | |  |
| 308 | What kind of toilet facility do members of your household usually use? | FLUSH OR POUR-FLUSH TOILET   1. Flush to a piped sewer system 2. Flush to septic tank 3. flush to a pit latrine 4. Flush to somewhere else 5. Flush, don't know where   PIT LATRINE   1. Ventilated improved pit latrine 2. Pit latrine with slab 3. Pit latrine without slab/open pit 4. composting toilet 5. bucket toilet 6. Hanging toilet/hanging latrine 7. No facility/bush/field | | |  |
| 309 | Do you share this toilet facility with other households? | 1. Yes 2. No | | |  |
| 310 | Including your household, how many households use this toilet facility? | 1. Less than 10 2. 10 or more 3. Don't know | | |  |
| 311 | Where is this toilet facility located? | 1. In own dwelling 2. In own yard/plot 3. Elsewhere | | |  |
| 312 | What type of fuel does your household mainly use for cooking? | 1. Electricity: 1. Yes, 2. No 2. Liquid petroleum gas: 1. Yes, 2. No 3. Natural gas: 1. Yes, 2. No 4. Bio-gas: 1. Yes, 2. No 5. Kerosene: 1. Yes, 2. No 6. Charcoal: 1. Yes, 2. No 7. Wood: 1. Yes, 2. No 8. Straw/grass: 1. Yes, 2. No 9. Agricultural crop: 1. Yes, 2. No 10. Animal dung: 1. Yes, 2. No 11. No food cooked in the house:1.Yes, 2.No | | |  |
| 313 | Is the cooking usually done in the house, in a separate building, or outdoors? | 1. In the house 2. In separate building 3. Outdoors 4. Other | | |  |
| 314 | Do you have a separate room which is used as a kitchen? | 1. Yes 2. No | | |  |
| 315 | Who is the owner of the house? | 1. Me 2. Rental 3. Family 4. Relative 5. Others (specify)_________________ | | |  |
| 316 | How many rooms in this household are used for sleeping? | --------Rooms | | |  |
| 317 | The main material of the roof of the house? | 1. Natural roofing (no roof, mud, and sod) 2. Rudimentary roofing (rustic mat/plastic shee, reed/bamboo, wood planks, and cardboard) 3. Finished roofing (metal/corrugated iron, wood, calamine/cement, ceramic tiles, roofing shingles) | | |  |
| 318 | The main material of the floor of the house? | 1. Natural floor (Earth/sand, dung) 2. Rudimentary floor (wood planks, and palm/bamboo) 3. Finished floor (parquet or polished wood, vinyl or asphalt strips/ plastic tiles, cement, ceramic tiles, carpet) | | |  |
| 319 | Does this household own any livestock, herds, other farm animals, or poultry? | Yes  No | | |  |
| 320 | How many of the following animals does this household own?  IF NONE, RECORD '00'.  IF 95 OR MORE, RECORD '95'.  IF UNKNOWN, RECORD '98'. | 1. Cows bulls_______________ 2. Other cattle______________ 3. Horses/Donkeys/Mules ____ 4. Camels________ 5. Goats _________ 6. Sheep _________ 7. Chickens/poultry ______ 8. Beehives ___________ | | |  |
| 321 | Do you have separate rooms for cattle? | 1. Yes 2. No | | |  |
| 322 | Does any member of this household own any agricultural land? | 1. Yes 2. No --------------------------------------- | | | 324 |
| 323 | How many hectares of agricultural land do members of this household own? | ___________hectares | | |  |
| 324 | Does any member of this household own: |  | Yes (1) | No (0) |  |
|  |  | 1. Electricity---------- 2. Radio--------------- 3. Television --------- 4. Non-mobile telephone---------- 5. Computer ---------- 6. Refrigerator ------- 7. Table---------------- 8. Chair --------------- 9. Bed with spring matters------------ 10. Electric mitad ----- 11. Kerosene lamp/pressure------ 12. Lamp--------------- |  |  |  |
| 325 | Does any member of this household own: |  | Yes  (1) | No  (0) |  |
|  |  | 1. Watch -------------- 2. Mobile phone------ 3. Bicycle------------- 4. Motorcycle/scooter- 5. Animal-drawn cart- 6. Car/truck ------------ 7. Boat with motor----- 8. Baggage ------------ |  |  |  |
| 326 | Does any member of this household have a bank account? | 1. Yes 2. No | | |  |
| 327 | Does any member of this household have a microfinance account? | 1. Yes 2. No | | |  |
| 328 | How often does anyone smoke inside your house?  Would you say daily, weekly, monthly, less often than once a month, or never? | 1. Daily 2. Weekly 3. Monthly 4. Less often than once a month 5. Never | | |  |

**Section III: Contraceptive/family planning methods related questions:** Ask the following questions one at a time and write the response on the space provided or encircle the appropriate response from the options listed.

| NO | Question | Response | | | | | Skip |
| --- | --- | --- | --- | --- | --- | --- | --- |
| 501 | Have you ever heard about family planning methods? | 1. Yes 2. No--------------------------------------------------- | | | | | 503 |
| 502 | Source of information  *Multiple answers are possible* | 1. Radio: 1. Yes 2. No 2. Television: 1. Yes 2. No 3. Newspaper message: 1. Yes 2. No 4. Community/ friends: 1. Yes 2. No 5. Health care providers: 1. Yes 2. No 6. Others (specify)________________ | | | | |  |
| 503 | What is family planning?  *Do not read the options, please* | 1. Planning the number of children, time, and space between children 2. Limiting the number of children 3. Stopping birth 4. The spacing of birth intervals 5. Do not know 6. Other (specify) _________________ | | | | |  |
| 504 | Which type of family planning methods did you know?  *Do not read the options, please* | 1. Oral contraceptive pills 2. Condom 3. Injectable 4. Implants 5. Intrauterine contraceptive device 6. Sterilization (male and female) 7. Calendar method 8. Periodic abstinence 9. Withdrawal(coitus interrupts) 10. Lactational amenorrhea method | | | | |  |
| 505 | What is/are the side effects of using family planning?  *Do not read the options, please* | 1. Heavy bleeding or irregular bleeding 2. Absence of menstrual cycle 3. Abdominal cramp 4. Head ach 5. Do not know 6. Other (specify) _________________ | | | | |  |
| 506 | Were you ever counselled about family planning methods? | 1. Yes 2. No | | | | |  |
| 507 | Do you currently utilize any of the family planning methods? | 1. Yes 2. No --------------------------------------- | | | | | 514 |
| 508 | If ‘yes’ to Q 5.6, Who used the method? | 1. Self 2. Couple (husband/wife) | | | | |  |
| 509 | What is the method you used currently?  *Multiple answers are possible* | 1. Oral contraceptive pills 2. Condom 3. Injectable 4. Implants 5. Intrauterine contraceptive device 6. Sterilization (male and female) 7. Calendar method 8. Periodic abstinence 9. Withdrawal(coitus interrupts) 10. Lactational amenorrhea method | | | | |  |
| 510 | Were you asked for payment for the family planning service? | 1. Yes 2. No | | | | |  |
| 511 | If ‘yes to Q 510, mention the payment in Birr | _______Birr | | | | |  |
| 512 | Were you counseled about, method options and side effects? | 1. Yes 2. No | | | | |  |
| 513 | Did you get the family planning method you choose? | 1. Yes 2. No | | | | |  |
| 514 | How far the nearest health institutions is from your home? | ________km or ______Minutes on foot | | | | |  |
| 515 | Is transportation available to the health facility? | 1. Yes 2. No | | | | |  |
| 516 | Did you use transportation to go to the health facility? | 1. Yes 2. No | | | | |  |
| 517 | If ‘No’ to Q 5.14, what is/are the reason? | Mention:________________________ | | | | |  |
| 518 | Is the health facility building accessible for you? | 1. Yes 2. No | | | | |  |
| 519 | Do you have permission from your partner (family) to use the method? | 1. Yes 2. No | | | | |  |
| 520 | Was the health facility (the provider) kept confidentiality? | 1. Yes 2. No | | | | |  |
| 521 | Was the health care provider giving the counselling or method alone by keeping your privacy? | 1. Yes 2. No | | | | |  |
| 522 | Do you have an intention to use the methods in the future | 1. Yes 2. No 3. Not sure | | | | |  |
| 523 | If 2 for Q 521, What is your reason | Mention:__________________________ | | | | |  |
| 524 | Reasons for not using family planning services | 1. Fear of side effects: 1. Yes 2. No 2. No information: 1. Yes 2. No 3. No permission from husband/wife1.Yes 2. No 4. No money for transportation 1. Yes 2. No 5. Distance to the health facility: 1. Yes 2. No 6. Waiting time to get service: 1. Yes 2. No 7. Not wanting to go alone: 1. Yes 2. No 8. Other (specify) _________________ | | | | |  |
| **The respondent's attitude toward family planning methods** | | | | | | | |
|  | **Variables** | **Strongly Agree** | **Agree** | **Do not know** | **Strongly Disagree** | **Disagree** |  |
| 525 | Do you think using FP makes women unhealthy? |  |  |  |  |  |  |
| 526 | Do you think pregnancy must be properly planned? |  |  |  |  |  |  |
| 527 | Do you think pregnancy spaced < 2 years should be avoided by using family planning methods? |  |  |  |  |  |  |
| 528 | Do you think the use of FP methods interferes with sexual relationships between husband and wife? |  |  |  |  |  |  |
| 529 | Do you think using modern FP methods causes anger from God? |  |  |  |  |  |  |
| 530 | Do you think FP methods result in infertility to getting pregnant later on? |  |  |  |  |  |  |
| 531 | Do you think women are more responsible than men for using modern FP methods? |  |  |  |  |  |  |
