# Supplementary material for: Contraceptive use among reproductive-age females with disabilities in central Sidama National Regional State, Ethiopia: a multilevel analysis
Source: PeerJ. 2023 May 12;11:e15354. doi: 10.7717/peerj.15354 (PMC10184657; doi:10.7717/peerj.15354)
Supplement: Supplemental Information 3 [file peerj-11-15354-s003.docx]

**AYIDDE KEEREHO!**

Xa’maanchu anino _________________________yaamameemmohu tenne taje gamba assate kaayyo atewa iilliteennae techo xaandoommo; tenne xiinxaallo xiinxallannohu rodiinke ZELAALEMI XEENAHO 3^ki^ digire Hawaasi yuniversitera Hikkiminnunna Fayyimmate kolleejera. Ani xa tenne yannara xiinxallo assanni noommohu 15-49 diri meereero noo bisu xe’ne noo meya beettora fayyimmate owaante afateeti Sidaamu Dagoomi Qoqqowi giddo

Xiinxallote umino’Haaro egenno kalanqe fayyimmansara bisu xe’ne noo amuwa sirote amanyooti aana xiinxallateeti. Xiinxallo hananfannihunno Onkoleessa 1-30/2014 geeshshaati. Kaayyo atera iillitinohe xa’matena, sumuu yoottaeha ikkiro harunsie:

**Xiinxallote Beeqqaancho Ikkakkira Mittu Qarri Di’’iillinohe!**

**Xiinxallote horo: xiinxallote beeqqo assootta daafira baattooshshu dino; kayinnilla, xiinxallote beeqqo assakki bisu aana xe’ne noo sirote orte amanyooti fayyimmara roorenkanni 15-49 diri mereero noo meyatera lowo irko afi’nanni.**

**FOJO AGARRANNI ORTE**

Xiinxallote beeqqo assatta yannara su’ma diboreessinanni horontanni. Ati dawarootta dawaro woleho fojo sayinse horontanni dikullanni. Dawarokki xiinxallote lowo qeechi noose; konni dafira, xiinxallote gumano mootimma noowa shiqinshanni daafira, mootimma adhitanno fayyimmate owaante woyyeessate loosira lowo eltooti. Kayinnilla, sumuu yaa hoogate qoosso agarantinote; ate fajjo gobbaanni mittoreno diborreesseemo.

Ane ledo keeshshineemmohu 20 xiqqessiraati!

Xiinxallote beeqqattara sumuu yaattae?

1- Ee 2 - Dee’ni

Sumuu yoottae daafira lowo geeshsha galaxxeemmo! Borri-xa’mo hanafi! If thethanks!

Dee’ni yootta daafira galaxxeemmo! Hanni gadachi xiinxallote beeqqo assitanno gede!

Fajjo borreessinoonni qoola xunse xiinxallote iillacha nabbawoomma/maciishshooma. Xiinxallote beeqqo asseemmohu umi’yanni sumuu yaatenniiti aye gadadishshi heerikkinniiti labbinoe dawaro dawareemmahu. Dawarata dawaro horontanni fojo agarrannita kawaanni qole qaaggisseemmohe. Konni daafira tenne xiinxallora beeqqo asseemma!

Xa’mamaanchu malaate_____________________________ barra _____________

Xa’maanchu su’ma: _________________________________________malaate_________

Xa’minoonni barri ----------- Aganunni --------------- /2014 Tophiyu kiiro garinni

Illaallisaanchu su’ma ___________________________________________ malaate________

Buunxoonni barra________________ /2014M.D.

Guutaaho 1

Diguutaho 2

Xiinxallote anni Su’ma: Zelalem Xeenawu

Iimeele: [abigiatenaw@gmail.com](mailto:abigiatenaw@gmail.com)

Bilibilla: +251-916415147

**Gamo I: Meessimmate Mayimma**

Konni woroonni noo xa’muwa sirote owaante qarri bisu xe’ne noo meyaata xa’minanni borri-xa’mo, Sidaamu Qoqqowi giddo.

Worada: ______________ Olluu koodde___________ Minu koodde ______________

| A.k | Xa’mo | Dawaro | | | Sai | |
| --- | --- | --- | --- | --- | --- | --- |
| 201 | Koo/tee | 1. Koo 2. Tee | | |  | |
| 202 | Dirikki me’’eho? | __________ dirooti | | | |  |
| 203 | Amma’nokki hiitteeti?  (dawarokki qoqqowi) | 1. Ortodokise/ Kiristaana 2. Kaatoolike 3. Pirotestaante /Kiristaana 4. Musiliime 5. Woleno (xawisi) __________ | | | |  |
| 204 | Hiikko ayiddeeti?/ Hiikko mineeti? | 1. Sidaama 2. Amaara 3. Oromo 4. Wolayitta 5. Guraage 6. Wole xawisi______________ | | |  | |
| 205 | Adhammete Gara | 1. Leexa ……………. 2. Adhaminoha 3. Tirroha/baxxinoha 4. Shiidhinota | | |  | |
| 206 | Teessokki? | 1. Quchumaho  2. Baadiyyete | | |  | |
| 207 | Olluu su’mi | : _____________________ti | | |  | |
| 208 | Aye ledo hee’ratta xa? | 1. Gashshaanni’ya ledo 2. Maate’ya ledo. 3. Calla’ya 4. Fiixi’ya ledo 5. Jaalla’ya/milla’ya ledo 6. Wole xawisi__________________ | | |  | |
| 209 | Rosu deerrikki? | 1. Nabbawanna borreessa didandiintanno 2. Xaaddote roso rossinote. | | |  | |
| 210 | Xa’mo 209 layinki doorsha ikkiro, me’e kifile geeshsha rossino? | ________kifile | | |  | |
| 211 | Loosikki doorshi mootimmanniha/loosu dinohe? | 1. Mootimmate loosaasinchooti 2. Loosu dinoe------------------- Dawarokki 2 kiiro ikkituro, 213 sai | | | 213 | |
| 212 | Loosikki dani? | 1. Mootimmate loosaasinchooti 2. Mootimmannita ikkitinokki uurrinshara 3. Maatete urrinshara. 4. Maatete uurrinsha ikkitinokkitera. 5. Hallanya | | |  | |
| 213 | Loosu noohe? | 1. Ee 2. Dee’ni ---------------------------------- dawarokki dee’ni ikkituro, 215 sai | | | 215 | |
| 214 | Loosikki qeechi maati? | 1. Ogimmate looso 2. Amma’note massagaancho. 3. Daddalaanchoho 4. Dandoo noote 5. Dandoo nookkite 6. Giwirinnu looso. 7. Wole xawisi______________ | | |  | |
| 215 | Ate laooshshinni, ati mitoricho hedatta dandookki hiittooti? | 1. Mulenni heda dandeemma 2. Hakeeshshi geeshshaati.. 3. Dibuxoomma | | |  | |
| 216 | Fayyimmate woowe noohe? | 1. Ee 2. Dee’ni ----------------------------------- dawarokki Dee’ni ikkituro, 301 sai | | | 301 | |
| 217 | Hiitti fayyimmate woowe noohe xawisi. | ______________________________ | | |  | |
| **Gamo II: Jajju woy Jirote Amadooshshe** | | | | |  | |
| 301 | Maatekki agganno waa afidhannohu hiikkiinniiti? | Baambu waa :   1. Mini giddo baambu waa 2. Hoowete afamanno baambu waa 3. Dagoomu tuqi horoonsi’ranno baambu waa 4. Umme fushshinoonni waa | | |  | |
|  |  | Agarooshshe:   1. Garunni agaraminoha 2. Garunni agaraminokkiha | | |  | |
|  |  | Buete waa:   1. Garunni agarroonnita 2. Garunni agarroonnikkita | | |  | |
|  |  | Daadanno Waa (Lagu/garbu/kofaminoha/xashshuwa/ waa) | | |  | |
| 302 | Maatekki sagale loosi’ratenna anga hayishshi’rate horoonsidhanno waa maminni afidhanno? | Baambu waa:   1. Mini giddo baambu waa 2. Hoowete afamanno baambu waa 3. Dagoomu tuqi horoonsi’ranno baambu waa 4. Umme fushshinoonni waa | | |  | |
|  |  | Agarooshshe:   1. Garunni agaraminoha 2. Garunni agaraminokkiha | | |  | |
|  |  | Buete waa:   1. Garunni agarroonnita 2. Garunni agarroonnikkita | | |  | |
|  |  | Daadanno Waa:  1. Lagu/garbu/kofaminoha/xashshu/ waa | | |  | |
| 303 | Way afamanno dargi hiikkooti? | 1. Meessi mini giddo  2. Meessi hoowe giddo  3. Wolu darginni | | |  | |
| 304 | Waa dirri’ne hinganni yanna mageeshsha adhanno? | 1. Daqiiqa/xu’eessa __________ 2. Dibuuxoommo/a | | |  | |
| 305 | Sa’uta lame lamala giddo way bae barra wo’ma keeshshino? | 1. Eewa 2. Dee’ni 3. Diqaagamannoe | | |  | |
| 306 | Way co’ichimma agarsiisate atewayinni agarrannire assoottori/tari no yite hedatta/o? | 1. Eewa 2. Dee’ni ------------------------------------- Dawarokki Dee’ni ikkituro, 308 sai | | | 308 | |
| 307 | Anganni way co’ichimma agara maa assa noohe?  Wo’manta hedo maareekki | 1. Gafe agatenni 2. Xagga woratenni 3. Hoccootunni ximbiiwaetnni 4. Waa ximbiimbanni meemonni (shaafa/shiimmaadda lubbuwa/etc.) 5. Arrishshote xawaabbinni xagisatenni 6. Mittowa kofatenni/ kuusatenni | | |  | |
| 308 | Maatekki duucha yannara hiittooha shumate mine horoonsidhanno? | **Wayinni xaadinsoonnita/ xaadinsoonnikkita**   1. Baamba fanne horoonsi’nannita 2. Baambaho xaaddino way maashine 3. Ummooni balera waa horoonsi’ra 4. Base baalate waa horoonsi’ratenni 5. Shumate mini waa hiissine horoonsi’nanniro diafoommo/a?   **Bale umme qixxeessinoonni shumate mine**   1. Ayyare e’anno shumate mine 2. Bale umme suudinsoonni kinchinni 3. Bale umme afoo tu’noonnikkita 4. Gate ha’rishshate horonsi’nanni shumate bale gede asse horoonsi’rate 5. Baaldete giddo horoonsi’neemmo 6. Gottiima shumate mine   7.Injiinowi dino/dubbu giddo/xawoho | | |  | |
| 309 | Tenne shumaate mini owaante woloota maate ledo horoonsidhinanni? | 1. Ee 2. Dee’ni | | |  | |
| 310 | Ate maate ledo mageeshshi mannaati konne shumate mine horoonsidhinannihu? | 1. 10 nni ajanno 2. 10 woy hakkuy ali 3. Afoommori/mari dino | | |  | |
| 311 | Hakku shumate mini afmannohu hiikkooti? | 1. Mini giddo 2. Meessi hoowe giddo 3. Wole dargaati | | |  | |
| 312 | Maatekki sagale loosi’rate hiittee gaaze horoonsidhanno? | 1. Korreentete wolqa 1.Ee 2 Dee’ni 2. Du’nantanno gaaze.Ee 2 Dee’ni 3. Kalaqamu gaaze 4. Addi addi keemikaalla.Ee 2 Dee’ni 5. Laamba .Ee 2 Dee’ni 6. Qitiissinoonni haqqe (kasale).1Ee 2 Dee’ni 7. Haqqe 1.Ee 2 Dee’ni 8. Kashee/hayissotenni 1.Ee 2 Dee’ni 9. Gidu damatenni 1.Ee 2 Dee’ni 10. Saadate obbinni 1.Ee 2 Dee’ni 11. Sagale diloosi’neemmo mini giddo 1.Ee 2 Dee’ni | | |  | |
| 313 | Sagale loosi’nannihu gallaniwaati, baxxitino kifileeti/ mini gobbaanniti? | 1. Mini giddooti 2. Baxxino mineeti 3. Barandahooti 4. Wolewaati | | |  | |
| 314 | Sagale loosi’rate horoonsidhinanni baxxino mini noo’ne? | 1. Ee 2. Dinonke | | |  | |
| 315 | Minu galtinannihu ayeho? | 1. Ko meessiho 2. Baante gallanniho 3. Maatenniho 4. Elunniho 5. Wole hee’riro xawisi_________________ | | |  | |
| 316 | Konni mini giddo gonxanni kifilla me’’e no? | --------Kifilla no | | |  | |
| 317 | Minu giddoydo iimiidi raga (korniise) mayinni biifinsoonniho? | 1. Kalaqamu haqqinni (biinfori dino, sabbunni,nna kandoonni sabbinni) 2. Calla anfi gede assinnoonniha (xu’minsoonnikki saattinni/plastic shara, shomboqqotenni/leemmichunni, haqqu xaawulinni, and komborsaatunni) 3. Xu’minsoonni korniise (siwiilunni/culku siwiilinni, haqqunni,alumineemetnni/simintotenni | | |  | |
| 318 | Mini uulliidi loosaminohu mayinniiti? | 1. Kalaqamunni noo gedeenni nooho (baatto/ bushshaho) 2. Safo tunge doogo hunnoonniho (haqqu xaawula, nna shomboqqotenni/ leemmichunni) 3. Xu’minsoonni mine (shakilunni) (haqqu xaawulinni xu’minsoonniha, uullano iimano shakila, simintotenni, alumineemetenni, uulla karranni shara(minxaafe) | | |  | |
| 319 | Minu anni / ati saadate hoshsho, baatto hawuurranni saadanna lukkuwa ceo noohe? | Ee  Dino | | |  | |
| 320 | Aliidi saada giddonni minu annihu mageeshshaati?  Mitturi nokki ikkiro (00) wori.  95 tu woroonna aleenni ikkiro (95) wori.  Kiiro anfonikkiha ikkiro(98) wori | 1. Saadanna bootta_______________ 2. Wole saada______________ 3. Farado/harre/gaangootta ____ 4. Gaala________ 5. Me’’e _________ 6. Ge’reewo _________ 7. Caacurre/ lukkuwa ______ 8. Diishshote koshsha | | |  | |
| 321 | Saada galtannowi baxxino dargi noo’ne? | 1. Eewa 2. Dinonke | | |  | |
| 322 | Maate’ne giddo loosi’nanni baatto afi’nohu no? | 1. Ee 2. Dino------------------------------------- Dawarokki Dino ikkituro, 324 sai | | | 324 | |
| 323 | Minu annira me’’e hekitaare ikkitannoti loossi’nanni baatto noosi? | ___________Hekitaare | | |  | |
| 324 | Konni mini maate umisinni afirinori hikuuriti? |  | Ee (1) | Dee’ni (0) |  | |
|  |  | 1. Korreente---------- 2. Raadoone ----------- 3. Telewishiine -------- 4. Mine silke---------- 5. Kompiitere ---------- 6. Mitore qiissanno udiinnichi ------- 7. Xarapheezzu ------- 8. Barcimu ------------ 9. Shiwote daallasi--- 10. Korreentete wolqanni sagale raisi’nanni mixashsho ----- 11. Laambunni/shaamu/ ------ 12. Faanoose------------ |  |  |  |  |
| 325 | Maatekki giddo togoo udiinni no? |  | Ee  (1) | Dee’ni  (0) |  | |
|  |  | 1. Tolobishiine -------- 2. Bilbilu------ 3. Shalleette------------ 4. Xexxerrisu------ 5. Saadate gaare- 6. Kaameelu/ hogowi kaameeli ------------ 7. Yowolo motor nooti--- 8. Sasu goommi baajaaje- |  |  |  |  |
| 326 | Maatekkira baankete akkowaante kiiro noonsa? | 1. Ee 2. De’ni | | |  | |
| 327 | Maatekki giddo shiimmaadda daddalu uurrinsha akkowaante kiiro noonsa? | 1. Ee 2. De’ni | | |  | |
| 328 | Maate’ne giddo sigaara wiliishshaahu barrunni me’’e higeeti?  Barrunni/lamalatenni/ aganunni/ dirunni / takkonta? | 1. Barrunni 2. Lamalatenni 3. Aganunni 4. Aganunni mitteege 5. Takkonta | | |  | |

**Gafa III: Maatete Damboowishsha Lainohunni Qixxaabbino Xa’muwa**

Woroonni shiqqino xa’muwara halaalaancho dawaro ikkitino doorte qoqqowi

| A.K | Xa’muwa | Dawaro | | | | | Sai |
| --- | --- | --- | --- | --- | --- | --- | --- |
| 501 | Maatete damboowishsha kayinsanna macciishshite egennootta/o? | 1. Ee 2. Dee’ni ---------------------------------------------- Dawarokki Dee’ni ikkituro, 503 sai | | | | | 503 |
| 502 | Mashalaqqe afi’rootta bu’a mamaati?  *Dawaro duucha ikkitara dandiitanno* | 1. Raadonetenni: 1. Eewa 2. Dee’ni 2. Telewishiinetenni: 1. Eewa 2. Dee’ni 3. Gaazeexunni: 1. Eewa 2. Dee’ni 4. Qooxxeessu dagoominni 1. Eewa 2. Dee’ni 5. Fayyimmate gargaraano: 1. Eewa 2 Dee’ni 6. Woleno (xawisi)________________ | | | | |  |
| 503 | Maatete damboowishshi maati?  *Doorha baala nabbabbooti* | 1. Qaaqquullu kiiro, xeertise ilatenna illanni darga balaxe mixi’rate 2. Qaaqquullu kiiro ajishate 3. Ila uurrisate 4. Ilate mereero noo yanna ledate 5. Dafoomma 6. Woleno xawisi | | | | |  |
| 504 | Hiikkonne maatete damboowishshi mixo hayyo hiittenne afootta/a?  *Doorsha Nabbabbooti!* | 1. Afuunni adhinanni kiniine / xagga 2. Kondome 3. Marfe 4. Dasaho worranni xagga 5. Otoottote worranni xagicho 6. Illannikki gede assate ) 7. Yanna kiiratenni 8. Godowu gatanno yanna qorophatenni 9. Labbaahu sirote guma gobbaanni hunatenna 10. Qaaqqoho unuuna duuchaage qansatenni | | | | |  |
| 505 | Maatete damboowishshinni daggara dandiitanno hori-gawajjo maati?  *Doorsha nabbabbooti!* | 1. Deerra sa’ino mundaa 2. Aganu mundee doycho ba’ne 3. Godowu game / xisso 4. Umu damuume 5. Dafoomma 6. Woleno xawisi _________________ | | | | |  |
| 506 | Maatete damboowishshi daafira amaalle egenninoonnihe? | 1. Ee 2. Dee’ni | | | | |  |
| 507 | Xaa yannara maatete damboowishshi mixo hayyo horoonsidhanni nootta? | 1. Ee 2. Dee’ni --------------------------------------- Dawarokki Dee’ni ikkituro, 514 sai | | | | | 514 |
| 508 | Xa’mote kiiro 506 nna 507, dawarokki ‘’Eewa’ ikkituro, assi’nanni gara ay kulihe? | 1. Ani umi’yanni 2. Aninna gashshaanni’ya | | | | |  |
| 509 | Muli yanna giddo hiittenne hayyo horoonsi’ritta?  *Duucha dawaro heedhanno* | 1. Afuunni adhinanni kiniine / xagga 2. Kondome 3. Marfe 4. Dasaho worranni xagga 5. Otoottote worranni xagicho 6. Illannikki gede assate ) 7. Yanna kiiratenni 8. Godowu gatanno yanna qorophatenni 9. Labbaahu sirote guma gobbaanni hunatenna 10. Qaaqqoho unuuna duuchaage qansatenni | | | | |  |
| 510 | Maatete damboowishshi daafira womaashsha xa’minoonnihe? | 1. Ee 2. Dee’ni | | | | |  |
| 511 | Aliidita xa’mote kiiro 510tdawarokki ‘Eewa’ ikkituro , birra xawisi | _______Birraati | | | | |  |
| 512 | Horoonsi’nanni doogganna hori-gawaajjo amaalle egenninoonnihe? | 1. Ee 2. Dee’ni | | | | |  |
| 513 | Ati ha’rootta maatete damboowishshi doogo afi’rootta? | 1. Ee 2. Dee’ni | | | | |  |
| 514 | Minikkira mulaahu fayyimmate mini mageeshsha xeerti’rannoho? | ________Km woy Lekkkate ------daqiiqi doogooti | | | | |  |
| 515 | Fayyimmate uurrinsha raga hodhishshu bado injiitannote? | 1. Ee 2. Dee’ni | | | | |  |
| 516 | Fayyimmate uurrinsha marate hodhishshu hasiissannohe? | 1. Ee 2. Dee’ni | | | | |  |
| 517 | Xa’mote kiiro 516te dawaro ‘Dee’ni’’ ikkituro, korkaatu maati? | xawisi:________________________ | | | | |  |
| 518 | Fayyimmate uurrinshara minnoonni ijaarri injaadoho? | 1. Ee 2. Dee’ni | | | | |  |
| 519 | Maatete damboowishshi mixo hayyo horoonsi’rate miilikki fajjannohe? | 1. Ee 2. Dee’ni | | | | |  |
| 520 | Fayyimmate mini loosaasine fojo maaxxaaareeti? | 1. Ee 2. Dee’ni | | | | |  |
| 521 | Fayyimmate mini ogeeyye doogga kultannohe wote foje maaxxeeti? | 1. Ee 2. Dee’ni | | | | |  |
| 522 | Tenne doogga horoonsi’rate konni albira ajuuja noohe ? | 1. Ee 2. Dee’ni 3. Afoommari dino | | | | |  |
| 523 | Xa’mote kiiro 522 te dawaro ‘Dee’ni’’ ikkituro, korkaatu maati? | xawisi:________________________ | | | | |  |
| 524 | Maatete damboowishsha horoonsi’rattakkihu mayraati? | 1. Iillishshara dandiitanno hori-gawajjo waajjeeti: 1. Ee 2. Dee’ni 2. Huwanyo hooggeennaeeti: 1. Ee 2. Dee’ni 3. Galte’ya fajjitannoekkihuraati 1.Ee 2. Dee’ni 4. Hodhishshaho woxu wolqa hooggeennaeeti 1. Ee 2. Dee’ni 5. Fayyimmate uurrinsha mini’yawiinni faffinoehuraati: 1. Ee 2. Dee’ni 6. Tara agadha giwisannoehuraati: 1. Ee 2. Dee’ni 7. Calla’ya ha’ra giwisannoehuraati: 1. Ee 2. Dee’ni 8. Woleno xawisi ) _________________ | | | | |  |
| **Dawaraasinete Lao Maatete Damboowishshi Mixo Doogga Lainohunni** | | | | | | | |
|  | **Babbadooshshe Xa’muwa** | **Lowo geeshsha sumuu yaa** | **Sumuu yaa** | **Diafoomma** | **Lowo geeshsha sumuu diyeemma** | **Sumuu diyeemma** |  |
| 525 | Maatete damboowishshi mixo amuwu fayyimmara bushate yite hedatta? |  |  |  |  |  |  |
| 526 | Godowatta yannara mixidhe egennootta? |  |  |  |  |  |  |
| 527 | Maatete damboowishshi mixo garinni 2 diri woro godowa dandiinannikkita afootta? |  |  |  |  |  |  |
| 528 | Maatete damboowishshi galtetenna gashshaanni qooda ikkinota baddine affinoonni? |  |  |  |  |  |  |
| 529 | Yannichu maatete damboowishshi doogga Maganu hanqo iillishshanno yite hedatta |  |  |  |  |  |  |
| 530 | Maatete damboowishshi ila hunanno yite hedatta? |  |  |  |  |  |  |
| 531 | Maatete damboowishshi annuwunni sao amuwaho yawo roortanno yite hedatta? |  |  |  |  |  |  |
